# Temporal Dynamics of Internal vs External Entrapment in Suicidal Ideation in Psychotherapy Outpatients: Prospective Longitudinal Cohort Study Using Ecological Momentary Assessment

**DOI:** 10.2196/81095

**Published:** 2026-08-04

**Authors:** Emmy Wichelhaus, Dajana Schreiber, Inken Höller, Thomas Forkmann

**Affiliations:** 1 Department of Clinical Psychology and Psychotherapy, Institute of Psychology, University of Duisburg-Essen, Universitätsstr. 2, Essen, 45141, Germany, 49 2011834502; 2 Department of Psychology, Institute for Mental Health and Behavioral Medicine, Health and Medical University, Düsseldorf/Krefeld, Germany

**Keywords:** EMA, entrapment, short-term risk dynamics, ecological momentary assessment, suicidal ideation

## Abstract

**Background:**

The Integrated Motivational-Volitional Model posits entrapment as a central driver of suicidal ideation (SI), yet little is known about how internal (IE) vs external entrapment (EE) unfolds in real time. Prior ecological momentary assessment (EMA) studies have generally treated entrapment as unidimensional and used sampling intervals of several hours, potentially obscuring rapid risk processes.

**Objective:**

This study examined (1) cross-sectional and longitudinal temporal associations between IE, EE, and SI across lags of 1‐3 prompts; (2) differences in immediacy of IE vs EE effects; and (3) whether the exact time interval (in minutes) between assessments moderates these associations.

**Methods:**

A total of 91 (62.2% female; M_age_=31.6, SD 10.6) adults initiating outpatient psychotherapy (convenience sample) completed a 7-day EMA protocol with 10 daily prompts (30‐120 min intervals). Participants provided informed consent, and ethical approval was obtained. A total of 5414 (85% compliance) observations were analyzed using multilevel models with maximum likelihood estimation (assuming missing at random). SI (4 items) and entrapment (IE, EE; 1 item each) were rated on 5-point scales. Lagged effects (up to ≈6 h) controlled for prior SI, and time intervals (in minutes) were tested as moderators.

**Results:**

SI varied primarily within persons (intraclass correlation=0.15). Cross-sectionally, both IE (est=0.16, 95% CI 0.08 to 0.24; *P*<.001) and EE (est=0.19, 95% CI 0.09 to 0.27; *P*<.001) were significantly associated with SI, explaining 25.3% of within-person variance (Quasi *R*²). Lagged analyses revealed distinct temporal patterns. At Lag 1 (30‐120 min), IE predicted increases in SI (est=0.13, 95% CI 0.09 to 0.18; *P*<.001), whereas EE did not (est=0.01, 95% CI –0.03 to 0.06; *P*=.60). At lag 2 (60‐240 min), EE predicted SI (est=0.05, 95% CI 0.01 to 0.10; *P*<.05), whereas IE was not significant. At lag 3 (90‐360 min), IE negatively predicted SI (est=–0.06, 95% CI –0.11 to –0.01; *P*<.05), while EE positively predicted SI (est=0.05, 95% CI 0.00 to 0.10; *P*<.05). Prior SI consistently predicted subsequent SI (est range=0.38‐0.42; *P*<.001). Time intervals did not moderate effects (*P* values>.12).

**Conclusions:**

Entrapment is a dynamic correlate of SI, with IE and EE showing distinct temporal patterns. IE has rapid, within-minute effects on SI, whereas EE shows delayed associations. This study is innovative in modeling minute-level dynamics using high-frequency EMA. Unlike prior unidimensional, lower-frequency designs, it captures ultra-short-term risk processes and clarifies temporally distinct pathways within the Integrated Motivational-Volitional Model and enhances theoretical precision regarding proximal vs distal drivers of SI. Clinically, results suggest that interventions may benefit from targeting IE in real time.

## Introduction

### Problem

Suicidal ideation (SI) is a significant clinical and public health concern and a potential precursor to suicidal behavior, with 9.2% of the global population experiencing some form of SI during their lifetime [1]. Understanding the mechanisms that underlie SI is crucial for the development of effective interventions. The Integrated Motivational-Volitional Model (IMVM) of suicidal behavior, proposed by O’Connor, and O’Connor and Kirtley [2,3], offers a comprehensive framework that proposes feelings of entrapment to be the central predictor of SI. Entrapment can be thought of as a psychological state in which individuals perceive themselves as trapped in a negative situation or set of circumstances that they believe cannot be escaped from [2]. The model suggests that when individuals experience entrapment, they are more likely to engage in SI as a way of escaping from this perceived inescapability, followed by increased intent, which may relate differently to psychological predictors and may exhibit different patterns of fluctuation over time [4-6].

Entrapment can be divided into two subdimensions, internal entrapment (IE) and external entrapment (EE). While EE refers to the perception of being trapped by external circumstances (eg, situational constraints and interpersonal demands), IE captures the experience of being unable to escape from one’s own thoughts, emotions, or mental states [7]. Recent clinical and psychometric findings based on the often-used Entrapment Scale (ES) [7] increasingly support a 2D structure. This indicates that IE and EE represent distinct yet related psychological phenomena with potentially different implications for SI [8-10]. Importantly, to date, this differentiation has not been systematically examined in real-time using ecological momentary assessment (EMA). EMA enables repeated assessments of participants within their everyday surroundings [11,12]. It allows for the recording of moment-to-moment fluctuations in psychological and behavioral variables and facilitates the analysis of relationships between these constructs both within and across different sampling points. In recent years, this approach has attracted considerable attention in the field of suicidology [13,14].

### Review of Relevant Scholarship

Although there is empirical support for the IMVM in various studies (eg, in other studies [15,16]), much of this research has been conducted with data from general population samples or in cross-sectional research designs. Few studies have explored the real-time dynamics of entrapment and its covariability with SI, especially in clinical samples. Longitudinal as well as real-time data are particularly valuable, as they allow for an examination of how these relationships evolve over time and under changing circumstances. In the past, it has been shown that both SI fluctuates over time [17] and that entrapment is a risk factor that is not stable over time, but fluctuates in its severity [18]. Accordingly, it might be assumed that these predictors also fluctuate in association with SI over time. Understanding whether entrapment leads to an increase in SI over time is an important step toward refining suicide prevention strategies. van Ballegooijen et al [19] examined the temporal relationships between defeat, entrapment, and SI in a clinical sample of individuals with SI and/or suicidal behaviors in the past month and the experience of at least one major depressive episode. Over seven days, participants reported their level of defeat, entrapment, and SI up to six times per day. Using multilevel vector autoregressive models, analyses revealed that higher momentary levels of entrapment predicted increased SI at subsequent time points across intervals of approximately 3, 6, 9, and 12 hours, with the association being particularly pronounced at shorter intervals around three hours, suggesting short-term dynamic processes. Furthermore, this study identified bidirectional associations between entrapment and SI. Entrapment appeared to function as a bridging variable linking defeat with SI, as stated in the IMVM. Overall, the findings underscore the role of entrapment as a central, temporally fluctuating risk factor in the suicidal process.

However, van Ballegooijen et al [19] have conceptualized entrapment as a unidimensional construct, thereby potentially overlooking meaningful differences in how IE and EE fluctuate over time and relate differently to SI in everyday contexts. Furthermore, no study to date has examined prediction intervals shorter than 3 hours between entrapment and SI—even though SI has been shown to fluctuate over very short intervals of minutes to hours (eg, the study by Hallensleben et al [20]), and the transition from suicidal intent to suicidal action often occurs within minutes [21-23], meaning that significant risk escalations in the suicidal process can occur very rapidly.

### Hypotheses, Aims, and Objectives

Against this background, this study aimed at investigating the longitudinal relationship between entrapment and SI across varying time intervals of minutes to hours in a sample of patients starting outpatient psychotherapy. By explicitly modeling IE and EE as separate constructs, the present examination offers a novel and clinically relevant extension of prior work. This approach enables a more fine-grained analysis of the short-term dynamics between different forms of entrapment and SI, and may ultimately help identify more precise intervention targets within the suicidal process. In line with assumptions of the IMVM, it is hypothesized that entrapment will predict SI both cross-sectionally and longitudinally. It is also hypothesized that IE will exhibit a stronger and more immediate predictive effect on SI than EE. Furthermore, it is examined whether the specific time interval (in minutes) has a moderating effect on the temporal relationship between entrapment and SI, such that the longer the time intervals, the weaker the temporal relationship.

## Methods

### Inclusion and Exclusion Criteria

Patients were included in this study if they gave written informed consent before the start of EMA assessments, endured a mental disorder according to *ICD-10* (*International Statistical Classification of Diseases, Tenth Revision*) [24], and started to receive treatment in the outpatient clinic. Exclusion criteria were an acute psychotic episode or an acute suicidal crisis at the time of recruitment. An acute suicidal crisis was defined as a state of imminent and high suicide risk characterized by (1) concrete suicidal thoughts, (2) the presence of a specific plan, (3) access to or availability of means, (4) inability to distance oneself from suicidal thoughts or intent, and (5) inability to engage in a reliable safety agreement. Suicidal thoughts emerging during this study were able to be addressed and treated within the context of the concurrent treatment.

### Participant Characteristics

The average age of participants was 31.6 (SD 10.6) years, with most participants being female (n=56, 62.2%). The demographic and clinical characteristics of the participants can be found in Table 1.

**Table 1.** Demographic and clinical characteristics of 91 participants. 7-day EMA^a^ examining entrapment and SI among adults initiating outpatient psychotherapy in Germany (study period: 2021‐2024). Diagnoses refer to *ICD-10*^b^ categories (eg, F32-33=depressive disorders; F40-41=anxiety disorders). Percentages are calculated relative to the total sample (N=91). Missing values indicate participants who did not provide information for the respective category.

	Values, n (%)
Sex	
Female	56 (62.2)
Male	27 (30.0)
Diverse	2 (2.2)
Missing	6 (6.6)
Diagnoses	
F2	1 (1.1)
F32-33	44 (48.4)
F34	5 (5.5)
F38	1 (1.1)
F40-41	13 (14.3)
F42	3 (3.3)
F43	17 (18.7)
F5	3 (3.3)
F6	8 (8.8)
F8	3 (3.3)
F9	6 (6.6)
Country of residence	
Germany	73 (80.2)
Missing	18 (19.8)
Mother tongue	
German	69 (75.8)
Other	4 (4.4)
Missing	18 (19.8)
Marital status	
Single	33 (36.3)
Partnered	27 (29.7)
Married	10 (11.0)
Divorced	3 (3.3)
Missing	18 (19.8
Employment status	
Employed	46 (50.5)
Unemployed	12 (13.2)
Retired	1 (1.1)
Stay-at-home parent	1 (1.1)
None of the above	13 (14.3)
Missing	18 (19.8)
Status of health	
Excellent	2 (2.2)
Very good	10 (11.0)
Good	38 (41.8)
Average	20 (22.0)
Bad	3 (2.2)
Missing	18 (19.8)
Exercise	
No	25 (27.5)
Yes	48 (52.7)
Missing	18 (19.8)
Smoking	
No	58 (63.7)
Yes	15 (16.5)
Missing	18 (19.8)
Alcohol	
No	32 (35.2)
Yes	41 (45.1)
Missing	18 (19.8)
Drugs	
No	61 (67.0)
Yes	12 (13.2)
Missing	18 (19.8)
Medication (including hormonal contraceptives)	
No	28 (30.8)
Yes	45 (49.5)
Missing	18 (19.8)
Lifetime mental disorder	
No	30 (33.0)
Yes	43 (47.3)
Missing	18 (19.8)
Current mental disorder	
No	15 (16.5)
Yes	58 (63.7)
Missing	18 (19.8)

a EMA: ecological momentary assessment.

b *ICD-10*: *International Statistical Classification of Diseases, Tenth Revision*.

### Sampling Procedures

Patient recruitment took place at the psychotherapy outpatient clinic of the University of Duisburg-Essen, Germany (convenience sampling). Data were collected as part of a larger project called “SYMNET” (“symptom networks of depression with special consideration of suicidality and interoception before and after cognitive behavioral therapy—an ecological momentary assessment study”). Recruitment of the sample took place between August 2021 and November 2024. Out of 212 patients eligible to participate, 99 declined participation or could not be contacted due to organizational reasons. A further 23 participants were excluded after participation because of compliance <50%. Thus, the data of 91 patients were analyzed in the final sample.

### Sample Size, Power, and Precision

An a priori power analysis [25] indicated that with a sample size of 90 participants and a completion rate of 85%, the current study would have sufficient power (>0.85) to detect at least medium-sized within-person effects. Thus, the sample size was sufficient to ensure that the nested structure of the data (ie, assessments at level 1 nested within participants at level 2) could be appropriately analyzed using multilevel models (MLMs). As the sample was recruited via convenience sampling, findings may not generalize to the broader outpatient population.

### Measures and Covariates

EMA data collection was conducted in a naturalistic setting, with participants receiving treatment as usual while simultaneously participating in this study. Participants were notified 10 times per day on seven consecutive days; prompts occurred randomly between 8 AM and 8 PM in intervals of 30‐120 minutes between prompts. At every prompt, participants were asked to rate their momentary level of passive (“at the moment, I have the feeling that life is not worth living” and “at the moment, there are more reasons for me to die than to live”) and active SI (“at the moment I want to die” and “at the moment I am thinking about taking my own life”) as well as IE (“at the moment, I feel like I’m in a deep hole that I can’t get out of”) and EE (“at the moment, I can’t see a way out of my current situation”) as the primary outcomes of interest in this study. Each construct was measured using a 5-point Likert scale (1: not at all to 5: extremely) with higher values indicating a higher level of the respective construct. A mean score was created for the four items assessing SI (total range 4‐20). For entrapment, both a mean score over both items (total range 2‐10) was calculated, as well as both items were used individually (IE: ranging from 1‐5, EE: ranging from 1‐5). Potential confounders might have been all moderator variables proposed in the IMVM [3]; however, it was only controlled for the autoregressive effect of SI on SI.

### Data Collection

EMA data collection was conducted via movisensXS [26] for the first 12.1% of the sample (until January 2022) and via the Catalyst app by MetricWire Inc [27] for the rest of the participants (since February 2022) on their private smartphones. The transition from movisensXS to Catalyst occurred due to technical reasons and the broader compatibility of Catalyst (eg, availability on both Android and iOS).

### Quality of Measurements

Participants completed 85% (SD 11.90) of the EMA assessments on average (minimum 50%, maximum 100%), resulting in 5414 valid observations (total proportion of missing EMA data: n=903, 15%).

### Instrumentation

The items have already been used and validated in previous studies and have been shown to have excellent psychometric properties [18,28].

### Conditions and Design

The dataset consisted of 70 (assessments at level 1) * 91 (persons at level 2)=6370 potential observations. The design represents a nonexperimental longitudinal EMA design with repeated within-person assessments nested within individuals.

### Ethical Considerations

The data collection procedure was in accordance with the Declaration of Helsinki [29] and was approved by the Ethics Committee of the Institute of Psychology, University of Duisburg-Essen, Germany (EA-PSY14/20/1709). Patients were included in the current study if they gave written informed consent before participation. To protect participant privacy and confidentiality, all data were anonymized or deidentified before analysis. All personal data were collected and stored separately from this study’s data. Data were stored securely in password-protected files, and only authorized personnel had access to the data. No images of identifiable individuals were recorded in this study. The participants received monetary compensation, which was graded according to their compliance, that is, the percentage of prompts answered in the EMA (see section Measures and Covariates): €50 was received for participation in the study, another €30 could be earned if an overall compliance of ≥80% was reached, and another €30 could be earned if an overall compliance of ≥90% was reached. A currency exchange rate of EUR €1=US $1.085 (average over the entire study period) was applicable.

### Data Diagnostics

To ensure that missing responses did not bias the analyses, multilevel logistic regression models were estimated to examine whether missingness of suicidal ideation (SI) was associated with prior SI, entrapment, or the time interval between prompts. These analyses indicated that missingness was not meaningfully related to prior SI or entrapment, and the effect of time between prompts was very small. Together, these results suggest that the data were likely missing at random (MAR). Accordingly, all MLMs were estimated using maximum likelihood, which yields unbiased parameter estimates under MAR [30,31].

### Analytic Strategy

MLMs were used to account for the hierarchical structure of the data, with repeated measures (level 1) nested within participants (level 2). MLMs allow for the estimation of both within-person and between-person effects, which is ideal for analyzing the momentary fluctuations in suicidal ideation (SI) and entrapment. The association between IE and EE at the within-person level was examined using the correlation of their person-mean-centered components. MLMs also accommodate time-varying predictors (EMA data) and individual differences in baseline levels of SI and entrapment. All MLMs were estimated using the lmer function from the *lme4* package in R (R Foundation). These models assume that the relationships between predictors and the outcome are linear, that residuals at each level are normally distributed and independent, and that residual variance is homoscedastic (ie, constant across levels of the predictors). Random effects (intercepts and, where included, slopes) are assumed to follow a multivariate normal distribution. Model fit was evaluated using maximum likelihood estimation for comparing fixed effects and restricted maximum likelihood for comparisons involving random effects. A total of 7 models (M0-M6) were computed for the analyses. For M5-M6, the optimization algorithm was explicitly set to “bobyqa” using the lmerControl function to ensure consistent convergence. In the moderation analyses of M5–M6, the moderator variable (time=duration between assessments in minutes) was divided by 100 before analysis, as the original scale values were too large and interfered with model convergence in R.

First, the baseline model (M0) was calculated to assess between-person variability in SI, with intraclass correlations (ICCs) used to indicate the proportion of variance explained at the between- or within-person level [32]. Next, the cross-sectional correlation between IE and EE (M1) and SI at t was computed, with predictors group-mean centered in R (centering within cluster [person], cwc). To assess multicollinearity, variance inflation factors (VIFs) were calculated for all predictors across models M1-M6. All VIF values were well below the conventional threshold of 5 (maximum VIF=2.80 [33]), indicating no evidence of problematic multicollinearity and supporting the simultaneous inclusion of IE and EE in the MLMs. Lagged effects of IE and EE (1‐3 lags; lag: M2, lag2: M3, lag3: M4) were then examined, controlling for lagged SI (all models) as well as all lags; for example, lag2 includes both t-1 and t-2 predictors, and lag3 includes t-1, t-2, and t-3 predictors. Specifically, lag1 refers to analyses from one prompt to the next (t-1 to t), lag2 from one prompt to the one after the next (t-2 to t), and lag3 from one prompt to the one after that (t-3 to t). Lags were defined by the sequential prompts, regardless of how much time had elapsed between prompts. If a prompt was missed, the lag was calculated from the last available prompt. To avoid “between-day” lags, the last value per day was not lagged, and all predictors were again group-mean centered (centering within cluster [person], cwc). Finally, within the t-(t-1) interval, the actual time gap in minutes is zoomed in on and treated as a moderator. The moderating effect of the time (interval) between two adjacent prompts (duration between assessments in minutes) on the relationship between IE and EE (at t-1, ie, lag1) and SI (at t) was investigated by including the interaction terms of IE (M5) and EE (M6) with time (duration between assessments in minutes) in the models.

Following common recommendations [34], deviance tests were conducted. Model selection was guided by the Akaike information criterion and the Bayesian information criterion, with random intercepts and slopes included when they improved model fit. As the focus was on fixed effects, random effects are reported only as the estimated proportion of positive slope [32].

## Results

### Recruitment and Participant Flow

**Figure 1.**
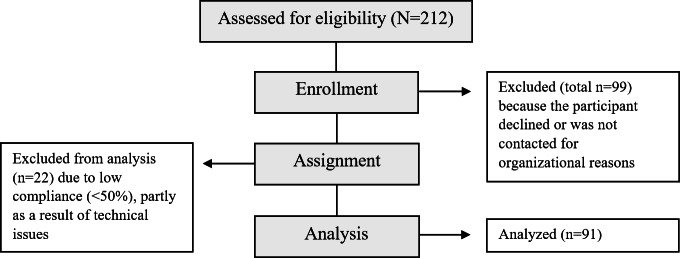
Participant flow diagram for this study: 7-day EMA investigating entrapment and SI among adults initiating outpatient psychotherapy in Germany (assessment period: 2021‐2024). EMA: ecological momentary assessment; SI: suicidal ideation.

Figure 1 shows the recruitment process and study flow in detail.

### Statistics and Data Analyses

#### Compliance and Missingness

Compliance across the EMA period was high, with participants completing, on average, 85% (SD 11.90%, range=50%‐100%) of prompts. To examine whether missing responses occurred systematically, multilevel logistic regression models were estimated predicting the missingness of suicidal ideation. Missingness was not predicted by prior SI (β=–0.01, SE=0.05, *P*=.86) or entrapment (β=–0.07, SE=0.04, *P*=.10). Although missingness was significantly associated with the time interval between prompts (β=–0.01, SE=0.00, *P*<.001), the effect size was very small, with each additional minute between prompts corresponding to only a 1% decrease in the odds of missingness (odds ratio=0.99). These results indicate that missing data were likely MAR.

#### Descriptive Statistics of the Main Study Variables

Overall, participants showed moderate levels of SI and entrapment, with between-person variability exceeding within-person variability (Table 2).

**Table 2.** Descriptive statistics for SI^a^ and entrapment variables. 7-day EMA^b^ examining entrapment and SI among adults initiating outpatient psychotherapy in Germany (study period: 2022‐2024). Observations: 5414 out of a maximum of 6370, missing percentage 15.22. All variables were measured via momentary self-report ratings (EMA) delivered 10 times per day.

Variable	Mean (SD)	Percentage of nonzero answers	Numbers with nonzero answers	ICC^c^ (95% CI)	rMSSD^d^ (SD, range)	Skewness	Kurtosis
SI	4.95^e^ (2.15)	36.7	55	0.85 (0.81 to 0.89)	0.74 (1.45, 0‐6.51)	2.89	9.28
Active SI	2.26^f^ (0.83)	24.4	29	0.80 (0.76 to 0.83	0.17 (0.46, 0‐2.74)	3.66	13.59
Passive SI	2.69^f^ (1.47)	35.8	51	0.83 (0.79 to 0.86)	0.42 (0.69, 0‐2.70)	2.42	6.16
Entrapment	3.82^g^ (2.19)	62.7	83	0.80 (0.76 to 0.83)	1.06 (1.20, 0‐7.04)	1.19	0.82
IE^h^	1.91^j^ (1.17)	55.4	75	0.76 (0.73 to 0.79)	0.39 (0.50, 0‐3.13)	1.19	0.68
EE^i^	1.92^j^ (1.15)	56.6	79	0.75 (0.72 to 0.78)	0.47 (0.48, 0‐2.35)	1.14	0.60

a SI: suicidal ideation.

b EMA: ecological momentary assessment.

c ICC: intraclass correlation.

d rMSSD: root mean square of successive differences.

e Mean of all four items, measured on a scale from 1 to 5, total range: 4‐20.

f Mean of both items, measured on a scale from 1 to 5, total range: 2‐10.

g Mean of both items, measured on a scale from 1 to 5, total range: 2‐10.

h IE: internal entrapment.

i EE: external entrapment.

j Measured on a scale from 1 to 5, total range: 1‐5.

#### Short-Term Variability

ICCs indicate that 15% of the variance in SI is because individuals differ in their mean SI (between-person variance), whereas 85% of the variance occurs at the within-person level (Table 2).

### Multilevel Analyses

#### Cross-Sectional Analyses

Table 3 presents the parameter estimates for the baseline model (M0) and the predictor model (M1) with SI as the outcome variable. In the baseline model (M0), the intercept was 4.95 (*P*<.001), indicating the average level of SI across all observations (grand mean). The ICC showed that 14.88% of the variance in SI was attributable to within-person differences, demonstrating variability in individuals’ mean levels of SI. In the predictor model (M1), both predictors—IE and EE—were significantly associated with SI. IE was positively associated with SI (est=0.16, *P*<.001), as was EE (est=0.19, *P*<.001). In addition, the estimates of the proportion of positive slopes (72.37% for IE; 71.48% for EE) suggest that these relationships were almost consistently positive across individuals. The 95% CIs for all estimates in M1 indicate a significant association between both IE and EE and SI, as the lower bounds of the CIs for both predictors are greater than 0, suggesting that these effects are robust and unlikely to be due to random sampling error. Finally, the quasi-*R*² indicated that adding IE and EE to the model explained 25.31% of the level-1 residual variance in SI compared to the baseline model (M0), demonstrating that momentary fluctuations in entrapment accounted for a meaningful proportion of within-person variation in SI.

**Table 3.** Parameter estimates for multilevel models with SI^a^ as the outcome variable (M0) and IE^b^ and EE^c^ as predictors (M1). N (level 2)=91. N (level 1)=5414.

	Fixed effects					Random effects
Model	Est.^d^	95% CI	SE^e^	*t* test (*df*)	*P* value	Slopes >0^f^ (%)
Predictors						
M0						
Intercept	4.95	4.52 to 5.29	0.21	23.97 (90.02)	<.001	N/A^g^
ICC^h^^,^ ^i^: 14.88% of the variance in SI is because individuals differ in their mean SI						
M1						
Intercept	4.95	4.58 to 5.40	0.21	23.97 (90.12)	<.001	N/A
IE	0.16	0.08 to 0.24	0.04	4.10 (72.26)	<.001	72.37
EE	0.19	0.09 to 0.27	0.05	4.16 (70.02)	<.001	71.48
Quasi *R*^2^: predictors of model 2 account for 25.31% of residual variance in active SI at level 1^j^						

a SI: suicidal ideation.

b IE: internal entrapment.

c EE: external entrapment.

d Est.: estimate (unstandardized regression coefficient).

e SE: standard error.

f Based on the assumptions of normally distributed slope coefficients, this value indicates the estimated percentage of slope coefficients that are positive.

g N/A: not applicable.

h ICC: intraclass correlation.

i Intraclass correlations indicate the proportion of variance explained at the between- or within-person level.

j Quasi *R*2 indicates the change in the residual variance in suicidal ideation when adding the models’ level 1 predictors compared to the baseline models.

#### Lagged Analyses

A moderate positive within-person correlation between IE and EE was found (*r*=.42), indicating that increases in IE tended to co-occur with increases in EE within individuals over time. Importantly, the magnitude of this association indicates that the constructs are related but not redundant, leaving substantial unique variance at the within-person level. This supports the assumption that IE and EE may exhibit partially distinct temporal dynamics, thereby justifying their simultaneous inclusion in the lagged MLMs. Table 4 reports parameter estimates from three lagged MLMs (M2-M4) examining temporal predictors of SI. All models included a random intercept and assessed whether lagged SI (lag1-SI) and lagged entrapment (IE and EE; lag1-IE and lag1-EE) predicted momentary fluctuations in SI across time. M2 evaluated the influence of lag1 variables. Lagged IE significantly predicted higher SI (est=0.13, *P*<.001). In contrast, lagged EE was not a significant predictor (est=0.01, *P*=.60), as its CI included zero. The model’s predictors accounted for 12.86% of the residual within-person variance in SI (marginal *R*²=0.1286), and the full model explained 87.44% of the total variance (conditional *R*²=0.8744). Model 3 examined lag2 effects while retaining lag1 predictors. Lag1-IE remained a significant positive predictor of SI (est=0.11, *P*<.001), whereas lag1-EE was not significant (est=–0.02, *P*=.52). At lag2, EE significantly predicted increases in SI (est=0.05, *P*.02), whereas lag2-IE did not (est=0.03, *P*=.15). The predictors accounted for 10.03% of within-person residual variance (marginal *R*²=0.1003), and the full model explained 87.73% of the total variance in SI (conditional *R*²=0.8773). M4 assessed lag3 effects while controlling for lag1 and lag2 predictors. Lag1-IE continued to significantly predict higher SI (est=0.12, *P*<.001), whereas lag1-EE remained nonsignificant (est=–0.02, *P*=.43). Neither lag2-IE (est=0.04, *P*=.15) nor lag2-EE (est=0.04, *P*=.08) significantly predicted SI. For lag3 effects, both predictors were significant: lag3-IE negatively predicted SI (est=–0.06, *P=*.021), whereas lag3-EE positively predicted SI (est=0.05, *P=*.046). Predictors in M4 accounted for 9.91% of within-person variance (marginal *R*²=0.0991), and the full model explained 86.94% of total variance (conditional *R*²=0.8694).

**Table 4.** Parameter estimates for lagged multilevel models with SI^a^ as the outcome variable and lagged IE^b^ and EE^c^ as predictors (M2-M4; RI^d^). 7-day EMA^e^ examining entrapment and SI among adults initiating outpatient psychotherapy in Germany (study period: 2022‐2024). N (level 2)=91. N (level 1)=5414. Lag1 refers to analyses from one prompt to the next (t–1 to t), lag2 from one prompt to the one after the next (t–2 to t), and lag3 from one prompt to the one after that (t–3 to t). Marginal *R*² values indicate the proportion of within-person residual variance explained by the level-1 predictors in each model; conditional *R*² values indicate the total variance in SI explained by the full multilevel model.

	Fixed effects				
Model	Est.^f^	95% CI	SE^g^	*t* test (*df*)	*P* value
Predictors					
M2					
Intercept	4.52	4.14 to 4.87	0.18	24.56 (92.13)	<.001
Lag1-SI	0.42	0.39 to 0.44	0.01	30.87 (4366.01)	<.001
Lag1-IE	0.13	0.09 to 0.18	0.02	5.98 (4358.12)	<.001
Lag1-EE	0.01	−0.03 to 0.06	0.02	0.53 (4364.40)	.60
Marginal *R*²: predictors of M2 account for 12.86% of the residual variance in suicidal ideation at level 1. Conditional *R*²: the full model accounts for 87.44% of the total variance in suicidal ideation across levels.					
M3					
Intercept	4.68	4.25 to 5.08	0.20	23.29 (110.30)	<.001
Lag1-SI	0.39	0.36 to 0.42	0.02	26.63 (3927.78)	<.001
Lag1-IE	0.11	0.07 to 0.16	0.03	4.56 (3910.92)	<.001
Lag1-EE	−0.02	−0.06 to 0.02	0.02	−0.65 (3889.32)	.52
Lag2-IE	0.03	−0.02 to 0.08	0.02	1.44 (3907.68)	.15
Lag2-EE	0.05	0.01 to 0.10	0.02	2.25 (3929.23)	.02
Marginal *R*²: predictors of M3 account for 10.03% of the residual variance in suicidal ideation at level 1. Conditional *R*²: the full model accounts for 87.73% of the total variance in suicidal ideation across levels.					
M4					
Intercept	4.56	4.17 to 4.93	0.20	22.37 (125.04)	<.001
Lag1-SI	0.38	0.35 to 0.42	0.02	24.67 (3551.89)	<.001
Lag1-IE	0.12	0.07 to 0.17	0.03	4.63 (3547.13)	<.001
Lag1-EE	−0.02	−0.07 to 0.03	0.03	−0.79 (3520.21)	.43
Lag2-IE	0.04	−0.01 to 0.09	0.03	1.46 (3529.95)	.15
Lag2-EE	0.04	−0.01 to 0.09	0.03	1.78 (3546.90)	.08
Lag3-IE	−0.06	−0.11 to −0.01	0.03	−2.30 (3553.41)	.02
Lag3-EE	0.05	0.00 to 0.10	0.02	2.00 (3527.72)	.046
Marginal *R*²: predictors of M4 account for 9.91% of the residual variance in suicidal ideation at level 1. Conditional *R*²: the full model accounts for 86.94% of the total variance in suicidal ideation across levels.					

a SI: suicidal ideation.

b IE: internal entrapment.

c EE: external entrapment.

d RI: random intercept

e EMA: ecological momentary assessment.

f Est.: Estimate (unstandardized regression coefficient).

g SE: standard error.

#### Time-Moderation: Very Short Time Intervals

Table 5 presents parameter estimates from two lagged MLMs (M5-M6) examining the moderation effect of minutes elapsed (time) between one prompt and the next (lag1) on the short-term effects of lag1-entrapment (t-1) and lag1-SI (t-1) on SI at t (ie, from one prompt to the next). In M5, lag1-IE (est=0.11, *P*=.003) significantly predicted higher SI, while the interaction between IE and the time interval between assessments (Time×lag1-IE) was not significant (est=–0.02, *P*=.57). Predictors in this model accounted for 31.85% of the residual within-person variance in SI, as indicated by the Quasi *R*² statistic. In M6, lag1-EE (est=0.04, *P*=.26) did not significantly predict SI, and the interaction term (time×lag1-EE) was likewise nonsignificant (est=–0.00, *P*=.91). Predictors in this model accounted for 31.18% of the residual within-person variance.

**Table 5.** Parameter estimates from lagged multilevel models (M5-M6) predicting momentary SI^a^ using time (in minutes) as a moderator. 7-day EMA^b^ examining entrapment and SI among adults initiating outpatient psychotherapy in Germany (study period: 2021‐2024). N (level 2)=91. N (level 1)=5414. Lag1 refers to analyses from one prompt to the next (t–1 to t).

	Fixed effects					Random effects
Model	Est.^c^	95% CI (est.)	SE^d^	*t* test (*df*)	*P* value	Slopes >0^e^ (%)
Predictors						
M5						
Intercept	4.69	4.33 to 5.04	0.18	25.62 (79.42)	<.001	N/A^f^
Lag1-SI	0.24	0.18 to 0.31	0.03	7.29 (44.27)	<.001	90.86
Lag1-IE	0.11	0.04 to 0.18	0.03	3.12 (42.78)	.003	75.67
Time x Lag1-IE	−0.02	−0.09 to 0.05	0.03	−0.57 (103.25)	.57	45.57
Quasi *R*^2^: predictors of model 5 account for 31.85% of residual variance in suicidal ideation at level 1^g^						
M6						
Intercept	4.66	4.33 to 4.99	0.16	29.54 (72.07)	<.001	N/A
Lag1-SI	0.23	0.17 to 0.31	0.03	7.40 (48.45)	<.001	90.53
Lag1-EE	0.04	−0.04 to 0.10	0.03	1.13 (72.13)	.26	58.87
Time x Lag1-EE	−0.00	−0.07 to 0.06	0.03	−0.11 (102.48)	.91	49.18
Quasi *R*^2^: predictors of model 6 account for 31.18% of residual variance in suicidal ideation at level 1^g^						

a SI: suicidal ideation.

b EMA: ecological momentary assessment.

c Est.: estimate (unstandardized regression coefficient).

d SE: standard error.

e Based on the assumptions of normally distributed slope coefficients, this value indicates the estimated percentage of slope coefficients that are positive.

f N/A: not applicable.

g Quasi* R*2 indicates the change in the residual variance in suicide ideation when adding the models’ level 1 predictors compared to the baseline models.

## Discussion

### Support of Original Hypotheses

This study examined the temporal dynamics of IE and EE in predicting momentary SI in adults beginning outpatient psychotherapy, using seven-day EMA. Consistent with hypotheses and the assumptions of the IMVM [3], both IE and EE were positively associated with SI cross-sectionally. Lagged analyses revealed a differentiated temporal pattern. IE showed consistent short-term effects, with higher IE at the previous prompt predicting increased SI at the subsequent prompt across models. In contrast, EE did not predict SI at the immediate next prompt but demonstrated delayed positive effects at longer lags (lag2 and lag3). Notably, a small negative association emerged for IE at lag3, whereas EE remained a positive predictor at this longer delay. Short-term moderation analyses further indicated that the time elapsed between adjacent EMA prompts did not significantly modify these associations. These findings highlight that momentary feelings of entrapment, particularly IE, are robust predictors of SI, with differential temporal patterns for IE and EE [3,35].

The present study complements prior research by zooming in on the very-short-term relations between entrapment and SI [19]. Specifically, the present results showed that IE significantly predicted SI at the shortest prediction interval (t–1, ie, from one prompt to the next), while EE did not; at longer prediction intervals, this pattern reversed, with EE exerting significant predictive effects only at t–2 and t–3. However, weak or nonsignificant associations cannot be due to a lack of power, as even with 49 prompts (lag 3), the power remains at >0.80 [36]. Thus, IE seems to exert its influence on SI more rapidly, whereas EE appears to require more time to manifest an effect. The entrapment—SI link at shorter prediction intervals appears to be primarily driven by IE—which might be triggered by cognitive processes such as rumination, self-devaluation, or emotional overwhelm [37,38]. EE, however, became predictive only over longer lags, suggesting that external stressors may exert slower, more gradual influences on SI [3,39]. One may speculate that the effect may further be attributable to external stressors increasing EE, further eliciting cognitive processes that subsequently trigger IE. Entrapment effects varied by lag and subcomponent: IE showed consistent short-term effects (lag1) and a small negative delayed effect at lag3, whereas EE demonstrated delayed positive effects emerging at lag2 and persisting at lag3.

### Similarity of Results

Previous studies reported mixed results as to whether entrapment should be deemed a 1D or 2D construct [7,9,35,40-42], with some suggesting that IE and EE may be of different importance for future SI. The conceptual differentiation of entrapment in the two subconstructs IE and EE is also supported by the findings of Lucht et al [43], who validated the motivational phase of the IMVM based on the cross-sectional data of a high-risk sample and demonstrated that IE and EE mediate the relationship between defeat and SI differently, with a greater effect size for IE, emphasizing the need to distinguish between the two subconstructs. Consistent with this, the longitudinal results of Höller et al [9] showed that IE was associated with SI as well as with a change in SI since the last measurement (t-1; 6 mo), while EE showed neither a significant association with SI cross-sectionally nor prospectively across all assessments spanning from six (t1), to nine (t2), to 12 months (t3). These results align with the present study’s finding suggesting closer relations between IE and SI than between EE and SI. IE might be more closely related to internal cognitive-affective processes and thus may exert a more direct and immediate influence on SI than EE.

The present results, however, contrast with those of van Ballegooijen et al [19], who reported a consistent predictive effect of entrapment as one construct on SI across all of the time intervals they examined (3‐12 h). The differences may be due to methodological factors. Both the present study and van Ballegooijen et al [19] reported relatively low within-person variance in self-reported SI among participants, which may have limited the sensitivity to detect effects [14,36]. However, they differed in how SI and entrapment were assessed. While van Ballegooijen et al [19] used a single item to assess SI, the current study used four items assessing both passive and active SI, introducing differences in both content and measurement reliability. Similarly, entrapment was assessed using different item sets: this study relied on established items from the ES, which had already been used in prior EMA research [18], whereas in van Ballegooijen et al [19], items were developed in collaboration with members of a lived experience research advisory panel, with the aim of ensuring ecological validity and relevance. These methodological differences may partially account for the divergent findings between the two studies, especially with respect to the divergent effects of IE and EE observed in the present analyses.

### Interpretation

The findings also underscore the importance of examining suicidal processes at very short time scales, particularly in light of current theoretical models that emphasize the potential for rapid and nonlinear escalation of suicidal risk. Descriptive frameworks such as the Suicide Crisis Syndrome [44], the Fluid Vulnerability Theory [45], or the Ambivalence Model of Suicidality [46] all share the assumption that suicidal crises can emerge quickly and are characterized by sharp, short-term increases in SI and intent. For instance, the Suicide Crisis Syndrome [44] posits a distinct clinical state marked by cognitive rigidity, frantic hopelessness, and emotional pain, which may precede suicidal acts by only a few hours or minutes. Similarly, the Fluid Vulnerability Theory [45] describes suicide risk as dynamic and context-sensitive, with acute risk states occurring against a backdrop of chronic vulnerability. The ambivalence model [46], in turn, conceptualizes SI as the result of a fluctuating interplay between the wish to die and the wish to live, which can rapidly shift in favor of action under internal or external strain. In line with these models, the present study’s finding that IE exhibits rapid, moment-to-moment associations with SI provides empirical support for the idea of fast-escalating suicidal states. This highlights the need to capture and understand not only the general association between entrapment and SI but also the rapid intraindividual transitions into acute suicidal states—which may remain undetected when longer intervals between assessments are used [14].

Taken together, these findings not only clarify how IE and EE unfold over time but also point toward broader implications for theory, assessment, and clinical intervention. As IE exerts rapid, moment-to-moment effects on SI, it can be assumed that the mechanisms driving suicidal thinking may be particularly tightly associated with internal cognitive–affective dynamics—and that these dynamics may shift fast enough to require real-time monitoring to be meaningfully captured [18,19]. Conversely, the more gradual influence of EE on SI implies that environmental pressures may accumulate and interact with internal states in ways that contribute to escalating risk, underscoring the importance of situating individuals within their lived contexts rather than relying solely on intrapsychic explanations [38,47]. More broadly, the temporal differentiation of IE and EE challenges models that conceptualize entrapment as a static or unified construct and instead supports frameworks that treat suicidal risk as inherently dynamic, multilayered, and temporally sensitive. This study advances the literature by modeling minute-level temporal dynamics of IE and EE using high-frequency EMA. In contrast to prior unidimensional and lower-frequency designs, this approach captures ultra-short-term risk processes and demonstrates temporally distinct pathways to suicidal ideation within the IMVM framework. By differentiating rapid, proximal effects of IE from the more delayed influence of EE, the findings refine the theoretical understanding of how suicidal states escalate in daily life. Importantly, identifying IE as an acute and near-immediate predictor of SI has direct clinical implications: it underscores the value of real-time monitoring and the potential of just-in-time adaptive interventions (JITAIs) that specifically target moments of heightened IE to interrupt short-term escalation of SI [48].

Strengths of this study include the use of EMA with high temporal resolution and multilevel modeling, which enabled robust within-person conclusions [36]. Of particular note is the moderator analysis using time as a high-resolution moderating variable in terms of minutes. Another strength of this study is the sample size. In the context of EMA studies, the results of the present study are based on a relatively large clinical sample (compare other studies [17,19,20]).

A limitation is the use of single items for both IE and EE measurement. Given that IE and EE have not yet been examined separately in EMA studies, a decision had to be made about which items to use for measurement. Items were taken from the German version of the ES [49]. Those items were chosen that had the highest factor loadings in their psychometric study at that time to ensure the items were most indicative of their respective constructs. However, 1-item measurements in EMA are potentially susceptible to validity limitations [50]. For example, the wording of the EE item, which uses the term “situation,” may be conceptually ambiguous, as it could be interpreted as either an external or internal situation, potentially confounding the construct with IE. Furthermore, the study by Wichelhaus et al [10] has since identified the highest factor loadings for other items. However, these results were not yet available at the time the data reported in the current study were collected. Given the potential conceptual overlap between the items, future research should draw on psychometric evidence to inform item selection, and both IE and EE should be assessed using multiple items.

Moreover, although previous research suggests distinguishing between passive and active SI [4-6], the reported analyses had to be based on a composite score including all four items on passive and active SI. As the outpatient psychotherapy sample showed lower variance in SI than might be found in a higher-risk population, such as inpatient settings, separating passive and active SI would have further limited variance in SI. Future studies with larger and more diverse samples could address this limitation by analyzing passive and active SI as distinct outcomes, ideally focusing on active SI, given its higher relevance for clinical intervention.

### Implications

We examined an outpatient sample without acute or severe suicidality and some diversity in terms of diagnoses. This may limit the generalizability of findings to higher-risk populations. Therefore, our findings open several avenues for future research. From a methodological perspective, the present results call for the integration of both IE and EE in EMA studies on entrapment. Moreover, more than one item per subconstruct is needed to examine factor structure and reliability in EMA [28]. To date, the psychometric properties of EMA-based IE and EE items remain untested, despite inconsistent factor structures reported in retrospective assessments of entrapment in paper-and-pencil questionnaires across languages [7,10,49,51,52]. This underscores the need for further validation of assessment instruments. In addition, the moderate within-person association between IE and EE observed in this study suggests that, although conceptually distinct, the constructs may share some common variance at the momentary level. This may partly reflect limitations in item-level measurement, particularly given the use of single-item indicators for each subcomponent [50]. Future research should therefore aim to refine the assessment of IE and EE in EMA contexts by using multi-item measures and systematically evaluating their psychometric properties, including within-person factor structure and discriminant validity. Such work will be essential to more clearly disentangle shared vs unique variance and to further clarify the extent to which IE and EE represent distinct dynamic processes over time. The present results may also motivate future research to examine further potential moderators—such as mood, coping skills, and social support—when modeling the within-person associations between entrapment and SI. Incorporating such moderators may clarify how emotional distress amplifies or how coping skills buffer the effects of IE and EE on SI [35,53-55].

While safety planning interventions remain a scalable and evidence-based foundation for suicide prevention [56], it might be beneficial to integrate EMA with passive data (eg, smartphone sensors) to capture dynamic shifts in risk factors (eg, activity, sleep, and speech) [57,58]. As safety plans provide individualized strategies for managing acute distress, they form the clinical cornerstone upon which JITAIs (eg, the study by Wang et al [59]) can build. JITAIs have the potential to deliver elements of safety planning dynamically—at moments when rising IE or EE indicates elevated risk—thereby potentially enhancing accessibility, engagement, and timeliness. JITAIs use real-time data to deliver personalized support during periods of elevated risk [48]. Their strength lies in responding quickly to behavioral and emotional changes—critical given the fluctuating nature of SI.

Future work should also consider examining which intervention components may be most effective in targeting IE-specific processes. Based on existing treatments, several approaches might appear promising: (1) cognitive behavioral therapy techniques such as cognitive reappraisal and behavioral experiments aimed at entrapment-related beliefs [60,61], (2) metacognitive therapy strategies (eg, detached mindfulness and challenging metacognitive beliefs) [62,63], (3) cognitive-behavioral suicide prevention approaches focusing on mapping and disrupting defeat-entrapment cycles [55,64], (4) acceptance and commitment therapy–based methods (eg, cognitive defusion, values clarification, and acceptance of internal distress) [65], and (5) emotion-regulation skills (eg, distress-tolerance strategies based on dialectical behavior therapy such as self-soothing, paired with emotional validation) [66,67].

The finding that entrapment, in particular IE, is related to increases in SI across very short time intervals underscores the potential of real-time monitoring of SI and timely interventions. The use of EMA allows for high-resolution tracking of moment-to-moment changes and can help detect acute risk periods early. From a therapeutic perspective, specifically targeting IE-related processes appears promising.

### Conclusions

In sum, differentiating internal and EE provides a nuanced understanding of temporal pathways to suicidal ideation, offering actionable insights for theory refinement, assessment, and timely clinical interventions in everyday life contexts. This study is innovative in several respects. First, by distinguishing between IE and EE at the within-person level using EMA methodology, the current study moves beyond traditional retrospective and cross-sectional approaches that dominate the entrapment literature [7,35]. This design enabled the examination of short-term temporal fluctuations in entrapment and SI as they unfold in daily life, thereby capturing dynamic psychological processes with greater ecological validity [68,69]. Second, whereas previous studies have often treated entrapment as a unitary construct or have focused predominantly on retrospective trait-like assessments [7,70], the present findings highlight the differential role of IE and EE, demonstrating that IE may represent a particularly salient proximal correlate of SI over short time intervals. This differentiation contributes to refining theoretical models of suicidality by suggesting that distinct forms of entrapment may operate through partially different mechanisms and may therefore require different clinical approaches [3,35].

The current study also extends the existing literature by integrating methodological, theoretical, and clinical perspectives. Methodologically, it emphasizes the importance of assessing both IE and EE simultaneously and illustrates the added value of EMA for investigating rapidly fluctuating suicide-related processes [20,71]. Theoretically, the findings contribute to contemporary models of suicide by supporting the view that entrapment is not static but dynamically linked to momentary changes in suicidal thinking, consistent with assumptions of the IMVM and related ideation-to-action frameworks [3,72]. Clinically, the results underscore the relevance of identifying and targeting IE-related processes in real time, particularly during periods of heightened psychological distress. In this regard, the findings provide an empirical basis for the future development of more precise and responsive intervention strategies, including JITAIs and EMA-informed suicide prevention approaches [48,59].

Importantly, the present findings also have implications beyond research settings. Real-time monitoring of IE and EE may help clinicians detect acute elevations in suicide risk earlier and tailor interventions more effectively to patients’ current psychological states [57,71]. Integrating such approaches into routine outpatient care, digital mental health applications, or suicide prevention services could improve the timeliness and personalization of support [48,59]. Furthermore, identifying momentary increases in IE may facilitate the delivery of targeted coping strategies before suicidal crises intensify, thereby potentially reducing barriers to help-seeking and improving continuity of care in everyday life [56,73]. Taken together, the present study advances the understanding of entrapment as a dynamic and clinically relevant process and highlights the potential of temporally sensitive assessment and intervention approaches to strengthen suicide prevention efforts.

## Abbreviations

EE: external entrapment
EMA: ecological momentary assessment
ES: Entrapment Scale
ICC: intraclass correlation
*ICD-10*: *International Statistical Classification of Diseases, Tenth Revision*
IE: internal entrapment
IMVM: Integrated Motivational-Volitional Model
JITAI: just-in-time adaptive intervention
MAR: missing at random
MLM: multilevel model
SI: suicide ideation
VIF: variance inflation factor
